# Spherical harmonics to quantify cranial asymmetry in deformational plagiocephaly

**DOI:** 10.1038/s41598-021-04181-z

**Published:** 2022-01-07

**Authors:** Jonas Grieb, Inés Barbero-García, José Luis Lerma

**Affiliations:** 1 Department of Cartographic Engineering, Geodesy and Photogrammetry, Universitat Politècnica de València, Camino de Vera, s/n, Building 7i, 46022 Valencia, Spain; 2 Department of Cartographic and Land Engineering, Higher Polytechnic School of Avila, University of Salamanca, Hornos Caleros 50, 05003 Ávila, Spain

**Keywords:** Paediatrics, Diagnosis, Applied mathematics

## Abstract

Cranial deformation and deformational plagiocephaly (DP) in particular affect an important percentage of infants. The assessment and diagnosis of the deformation are commonly carried by manual measurements that provide low interuser accuracy. Another approach is the use of three-dimensional (3D) models. Nevertheless, in most cases, deformation measurements are carried out manually on the 3D model. It is necessary to develop methodologies for the detection of DP that are automatic, accurate and take profit on the high quantity of information of the 3D models. Spherical harmonics are proposed as a new methodology to identify DP from head 3D models. The ideal fitted ellipsoid for each head is computed and the orthogonal distances between head and ellipsoid are obtained. Finally, the distances are modelled using spherical harmonics. Spherical harmonic coefficients of degree 2 and order − 2 are identified as the correct ones to represent the asymmetry characteristic of DP. The obtained coefficient is compared to other anthropometric deformation indexes, such as Asymmetry Index, Oblique Cranial Length Ratio, Posterior Asymmetry Index and Anterior Asymmetry Index. The coefficient of degree 2 and order − 2 with a maximum degree of 4 is found to provide better results than the commonly computed anthropometric indexes in the detection of DP.

## Introduction

Deformational Plagiocephaly (DP) is a deformation of the infant's skull due to positional causes. It consists of a flattening of an area of the head, resulting in asymmetry^1^. The prevalence of DP has been studied by different authors, reporting a percentage of affected infants ranging from 22.1^2^ to 46%^3^, with some intermediate values as the 37.8% reported by Ballardini et al.^4^.

Although the consequences of DP have lengthily been considered merely esthetical, during the last years it has been stated by some authors as an indicator of developmental delay risk^5–7^.

Positional cranial deformation is usually measured by paediatricians and neurosurgeons using callipers (cephalometers) and measuring tape to extract different indexes. The most common indexes are the Cephalic Index (CI), the Asymmetry Index (AI) and the Oblique Cranial Length Ratio (OCLR)^8^. The accuracy of calliper measurements is highly questioned^9,10^. The obtainment of accurate measurements with the baseline methods on the patients is complicated even for experts^11^ as infants do not usually cooperate and get nervous during the measurements.

Nowadays, the use of 3D models to evaluate cranial deformation is becoming more common. However, due to their high cost, they are not widely implemented in clinical practice.

The methodologies to obtain three-dimensional (3D) models include low-cost tools^12^ and, more commonly, setups of cameras and scanners^13,14^. Radiological tests, such as Computed Tomography and Magnetic Resonance Imaging are considered the gold standard but they are also highly invasive and costly. For these reasons, their use is very limited. Even in these cases, the measurements carried out on the 3D model are very similar to those manually carried out by doctors namely paediatricians and paediatric neurosurgeons, and limited to some indexes. Some authors have applied different mathematical processes to the classification and evaluation of cranial deformation from 3D models. Some examples are Principal Components^15^, the Root Mean Square differences between head quadrants^16^, kernel density estimation^17^, and deep learning for craniosynostosis diagnosis^14^. Furthermore, cranial deformation has been assessed by comparing the measured head shape to a fitted tri-axial ellipsoid which was considered as an ideal shape for a non-deformed cranium^18^.

The asymmetrical flattening of the back of the head caused by DP usually leads to an asymmetrically fitted ellipsoid (i.e. one side of the back of the head is outside the ellipsoid while the other side is inside). Although the differences to an ideal ellipsoid allow a good visual assessment of the deformation, no indexes have been developed that indicate the presence of deformation and DP in particular.

Spherical harmonics are commonly used to quantify and model irregular shapes in different fields, such as the shape of the Earth’s geoid in geodesy^19–21^, gravitational fields in geophysics^22^, bathymetry^23^, different biological structures^24^ or surfaces for meshing^25^.

In the field of medicine, spherical harmonics are frequently used as part of recent efforts to develop computer-aided, non-invasive methodologies to predict tumour growth behaviour, on the hypothesis that malignant tumours tend to have more irregular shapes in comparison to benign tumours. An example of this application is the use of spherical harmonics for the development of a computer-aided methodology for the diagnosis of thyroid cancer^26,27^. The contrast between the approach used in the proposed paper for cranial asymmetry quantification and the approach for diagnosis of malignant tumours can be of great value, due to the different spherical harmonics approach undertaken herein. Other medical applications of spherical harmonics include neuroscience, where they have proven to be useful for the segmentation of brain white matter fibres into bundles^28^, for modelling of the brain shape^29^ and for registration of brain hemispheres^30^.

In this work, the differences between the real head and an ideal ellipsoid have been extracted for different heads and modelled using spherical harmonics. From a mathematical viewpoint, the infant’s head can be considered equivalent to a tiny partial geoid. Therefore, spherical harmonics will be applied in this paper to determine its reliability to assess asymmetry in cranial deformation.

In this study, the weight of the spherical harmonic which best reflects the asymmetrical flattening of the head has been identified. The parameter has been evaluated in comparison with commonly used indexes that quantify plagiocephaly and it is proposed as an indicator for the automatic detection of this type of deformation in cranial 3D models.

## Material and methods

All research was performed in accordance with relevant guidelines and regulations. The data used in this research was collected as part of a project approved by the medical ethical review board (Comité de Ética de la Investigación con medicamentos) of the Hospital Universitario y Politécnico La Fe, Valencia, Spain (n. 2019/0217). All data was anonymized and informed consent was obtained from parents or legal guardian for each patient.

**Figure 1:**
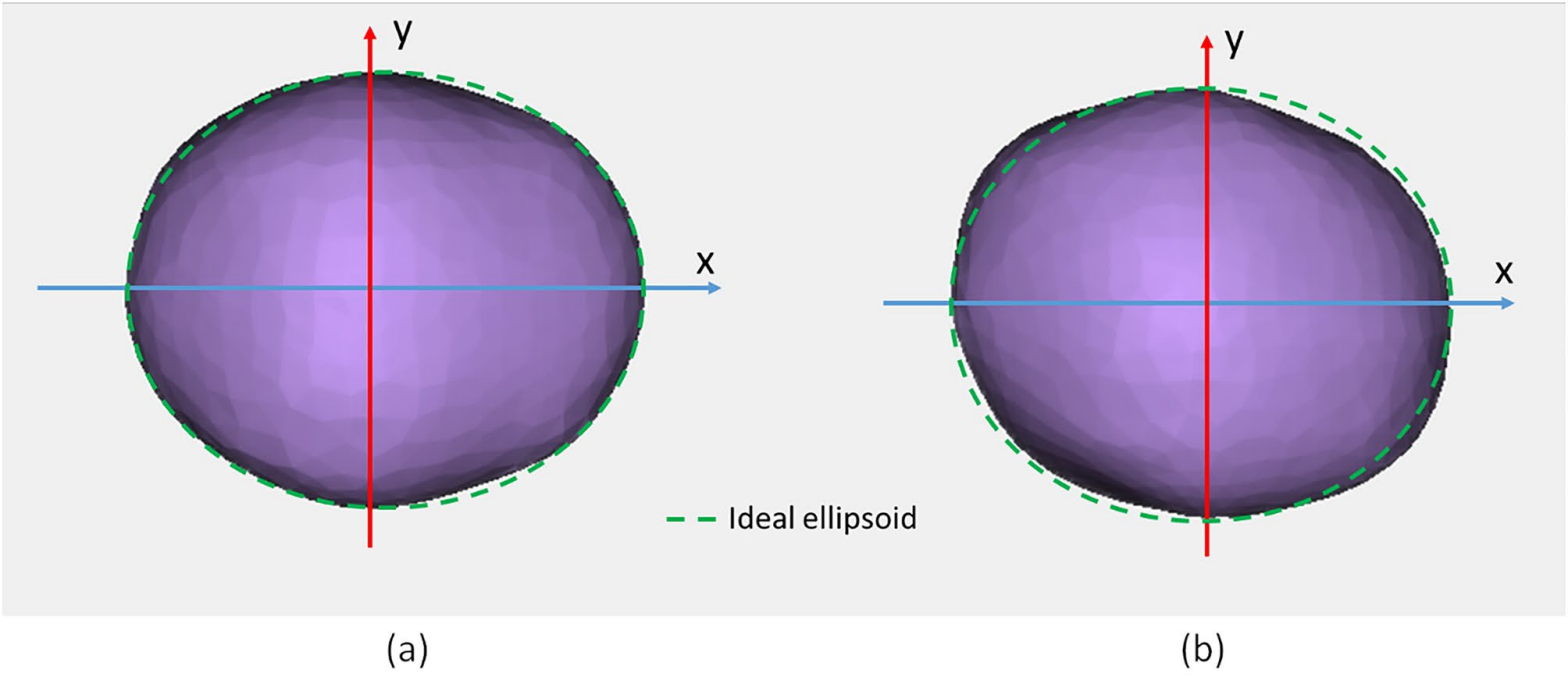
Visualization of lheads’ 3D model with the ideal fitted ellipsoid section on top: (**a**) regular patient (ID: H1, Table 1); (**b**) patient with DP (ID: P01, Table 1).

A total of 18 three dimensional models (Fig. 1) of infants' heads were obtained during normal neurosurgery consultation. Some of the infants were in different phases of treatment for cranial deformation by paediatrics neurosurgeons while others were control patients with no diagnosed deformation.

For each infant’s 3D model, different anthropometric indexes of cranial deformation were extracted automatically using the patented smartphone-based photogrammetric solution that just requires a coded cap fitting on the infant’s head^31^.The Photogrammetric Medical Deformation Assessment Solutions (PhotoMeDAS, https://photomedas.eu/) is based on the previous patent to automatically achieve the head’s 3D model and deformation indexes^12^. Later, the ideal ellipsoid (Fig. 1) was computed for each head and the distances to this ideal shape were modelled using spherical harmonics. For this latter purpose, an in-house Python script was developed which runs the Spherical Harmonic Tools (SHTools) library^32^ to fit the spherical harmonic coefficients to the distances. SHTOOLS/pyshtools is a Fortran-95/Python open-source library that can be used to perform spherical harmonic transforms and is available with a permissive licence at GitHub (https://github.com/).

### The creation of the models

The data acquisition was carried out using the low-cost PhotoMeDAS solution^12^ in approximately 2 min per patient. Each model was composed of approximately 530 points, evenly distributed over the head surface.

Each model was registered to a known coordinate system using 3 points manually identified by the doctor or technician during the data acquisition. These points correspond to the preauricular points and the point between the eyes. The registration methodology was established to facilitate the data acquisition by medical professionals (minimum number of points manually identified) and reduce errors and variability (points easily identifiable, even by non-experts)^33^.

The X-axis is defined by the point between the eyes and the mid-point between preauricular points. The Y-axis is perpendicular to the X-axis and adjusted to be as close as possible to both preauricular points.

The cranial models are all centred at the origin and facing towards the positive direction of the X-axis. Therefore, all cranial models are roughly divided longitudinally by the X-axis (i.e. following the anterior–posterior axis) and divided by the Y-axis from ear to ear (Fig. 1).

### Deformation indexes computation

The deformation indexes are usually derived from measurements carried out by doctors (i.e. paediatricians or neurosurgeons) using callipers and measuring tape. The manual measurements are standardized, and usually include cranial perimeter, oblique diameters, head width and head length^10,34,35^. The reproducibility of manually extracted heads measurements relies on the standard position of the head^10^ and the interuser reliability can be low^36^. In this case, the measurements were automatically extracted from the 3D model using the PhotoMeDAS tool^12^, avoiding any human error and blunders while writing or typing the analogue manual measurements.

**Figure 2:**
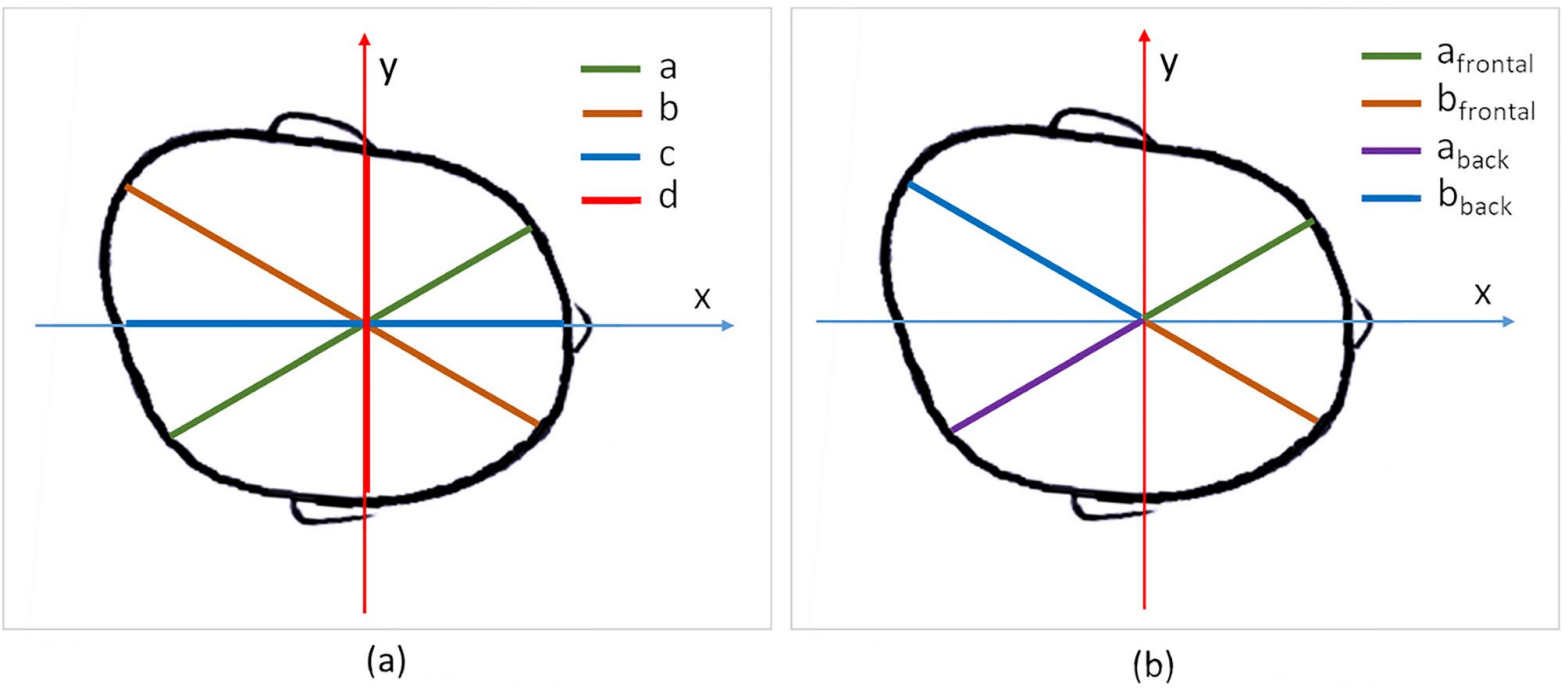
Visualization of measurements taken for the computation of the anthropometric deformation indexes: (**a**) traditional measurements undertaken with conventional analogue solutions; (**b**) extra measurements extracted automatically with PhotoMeDAS.

The measurements carried out for this study include oblique distance front-left to back-right (a), oblique distance front-right to back-left (b), head length (c), head width (d) and cranial perimeter (CP) (Fig. 2a). The CP is obtained as the maximum perimeter of the head contained in a plane parallel to the Y-axis. The oblique measurements are computed at a ± 30º angle with X-axis in the plane of the maximum perimeter. The head length is computed as the maximum length in the X–Z plane and the width length is the maximum distance in the direction of the Y-axis.

Linear measurements (a & b) were also divided as frontal and back, yielding a_frontal_, a_back_, b_frontal_ and b_back_ (Fig. 2b). The division of frontal and back parameters is not a common approach as these parameters are impossible to extract by manual calliper measurements. However, their extraction is automatic from the 3D model using computer-graphics in PhotoMeDAS. Besides, they allow the computation of two new indexes, Anterior Asymmetry Index (AAI) and Posterior Asymmetry Index (PAI), that are easy to determine, and useful, as it will be demonstrated later. From a clinical viewpoint, they can help doctors to realise where the DP occurs, either in the frontal part or in the back (parietal/occipital) part, without any compensation as happens with the classical a & b oblique linear measurements.

For each model, the following indexes were extracted:

- Cranial Perimeter, CP
- Cephalic Index, CI: $\left(\frac{c}{d}\right)$
- Cranial Vault Asymmetry Index, AI: ($a-b$), where a and b are the diagonals at ± 30º from the longitudinal axis of the head (Fig. 2a)
- Oblique Cranial Length Ratio, OCLR: $\left(\frac{a}{b}\right)*100$
- Anterior Asymmetry Index, AA: I ${a}_{frontal}-{b}_{frontal}$ (Fig. 2b)
- Posterior Asymmetry Index, PAI: ${a}_{back}-{b}_{back}$

While the AI and the OCLR are commonly calculated by doctors, the Anterior and Posterior Asymmetry Indexes (AAI and PAI) cannot be calculated from calliper and tape measurements. These new indexes are able to measure the deformation for the back and frontal parts of the head only. Thus, AAI and PAI are not affected by the compensation of the head shape that may occur in some cases of plagiocephaly (and especially in some type of craniosynostosis) and which prevents the AI to properly represent the infant’s cranial deformation^37^.

### Classification of the patients

The 18 patients were initially classified as healthy of DP depending on whether they had an earlier diagnosed plagiocephaly by the paediatrician neurosurgeons.

For each patient, all the asymmetry indexes (AI, AAI and PAI) and OCLR were computed. Normal values are considered below 4 mm for asymmetry and 94–106% for OCLR^38^. Any patient with an index not matching the normal values was considered to have positional plagiocephaly.

One of the patients initially classified as healthy or with regular shape based on visual assessment (ID 8 in Table 1) resulted eventually in the range of values of cranial deformation and was, therefore, moved to the DP group.

**Table 1:** Deformation parameters and classification for the patients.

Patient ID	CP (mm)	CI (%)	CVAI (mm)	AAI (mm)	PAI (mm)	OCLR %	Initial classification	Final classification
H1	435	85	− 2	− 2	0	101	Healthy	Healthy
H2	487	84	1	2	− 1	99	Healthy	Healthy
H3	480	81	2	0	2	99	Healthy	Healthy
H4	423	81	− 2	2	− 4	101	Healthy	Healthy
H5	453	78	− 3	1	− 3	102	Healthy	Healthy
H6	493	78	4	1	1	98	Healthy	Healthy
H7	424	81	− 2	0	− 2	101	Healthy	Healthy
H8	404	85	3	2	2	98	Healthy	Healthy
P1	401	84	− 4	− *5*	1	103	DP	DP
P2	410	84	− *12*	− 2	− *10*	*108*	DP	DP
P3	376	89	− *9*	− 3	− *6*	106	DP	DP
P4	415	90	− *10*	− 3	− *8*	105	DP	DP
P5	405	89	*5*	*9*	− 4	97	DP	DP
P6	478	87	− *13*	− 4	− *9*	*108*	DP	DP
P7	503	76	*9*	1	*8*	94	DP	DP
P8	439	87	− *8*	− 4	− 4	104	DP	DP
P9	372	79	4	*9*	− 4	98	DP	DP
P10	460	86	*12*	*5*	*7*	*93*	Healthy	DP

In italic values outside the normal threshold.

All patients had Cephalic Indexes in the regular range of 75–95%. Therefore, it can be stated that all 18 patients were not affected by scaphocephaly or brachycephaly^38^.

### The spherical harmonics solution

The infant’s head 3D models were assessed by computing the orthogonal distances to a fitted ellipsoid by spherical harmonics. The best-fitted ellipsoid is considered as the ideal cranial shape and is therefore used as the spherical reference surface^18^. In contrast, the true surface of the infant’s head 3D model can be considered as a geoid from a mathematical viewpoint.

To calculate the spherical harmonics for a single cranium the following steps were taken:

1. Calculation of the best-fitted ellipsoid for the given cranium.
2. Calculation of the orthogonal distances between the calculated ellipsoid and the real surface of the cranium.
3. Performing a spherical harmonics expansion to obtain the coefficients which are fitted to model the orthogonal distances.

The following lines present the mentioned three steps in detail.

#### Best fitting ellipsoid

A triaxial ellipsoid has been described before as the ideal shape to represent a cranium^18^. Therefore, the triaxial ellipsoid which is best adjusted to the 3D point cloud already available from the 530 cap points is computed with the least-squares estimation as in^39^. The best-fitted ellipsoid is calculated for each head individually and serves as a spherical reference surface on which the spherical harmonics will be computed.

The orientation of the ellipsoid is aligned with the coordinate system of the input data (as displayed in Fig. 2). Therefore, the ideal ellipsoid is symmetric with respect to the longitudinal axis of the infant’s head and it is not affected by the asymmetry of the head.

#### Orthogonal distances to the ellipsoid

In order to model the cranial shape with spherical harmonics, it is required to map every position on the surface of the cranium to a unique position on the surface of the ellipsoid, which serves as the reference surface.

The orthogonal distances for every point of the infant’s head 3D model are computed to the surface of the fitted ellipsoid as described by Bektas^40^.

#### Calculation of the spherical harmonic coefficients

For every point on the reference ellipsoid given by the spherical coordinates (θ, φ) the variation to the true infant’s head surface is now given by the associated orthogonal distance. Finally, these orthogonal distances are modelled with a linear combination of weighted spherical harmonics. A real spherical harmonic *Y*_*l*_^*m*^ and its respective coefficient (or weight) *f*_*l*_^*m*^ refer to a degree *l* and order *m* where (− *l* ≤ *m* ≤ *l)*. A linear combination of all real spherical harmonics up to a defined maximum degree *l*_*max*_ can approximate an arbitrary function on the sphere^32^. Based on this, we model every point $P\in {R}^{3}$ of the true cranium, represented by the 3D point cloud O, as follows:

1 $$P\in O={Q}_{P}=\left(\theta ,\Phi \right)=E\left(\theta ,\Phi \right)+\widehat{v}\left(\theta ,\Phi \right)\cdot SH\left(\theta ,\Phi ,{l}_{max}\right)+\varepsilon $$

where Q_P_ is the projected P on the surface of the best-fitted ellipsoid E expressed in spherical coordinates (θ, φ), $\widehat{v}$(θ, φ) is the unit vector orthogonal to the surface of the ellipsoid at Q_P_, and ε is the error of the model.

**Figure 3:**
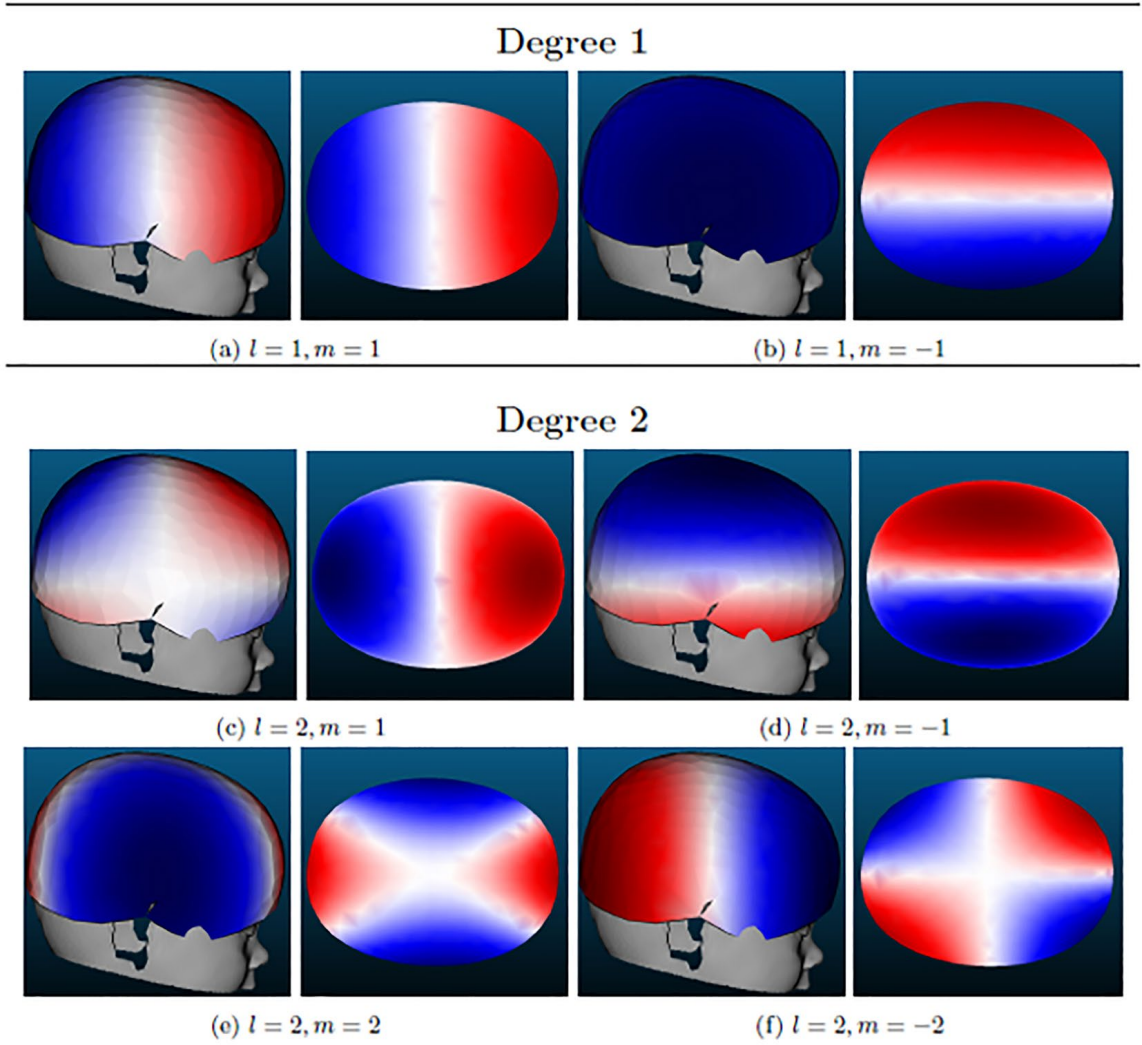
Visualization of spherical harmonic functions of degree l = 1 and l = 2 (excluding those of order m = 0) on the right-hand side (lateral) view and the top view. Red and blue symbolize positive and negative function values, respectively.

SH(θ, φ, l_max_) refers to the linear combination of weighted spherical harmonics. The accuracy of this mathematical model depends on the chosen maximum spherical harmonic degree, l_max_. The higher l_max_ is chosen, the more spherical harmonics are included in the linear combination and thus the more detailed becomes the reconstructed cranial shape. This is due to the property of spherical harmonics having l-|m| zero crossings in the latitudinal and 2 *|m| zero crossings in the longitudinal direction^32,41^. Thus, with an increasing degree l the surface of the sphere is divided into more and smaller sections. Therefore, with an increasing l_max_ a linear combination of spherical harmonics can represent more details on the surface of a 3D object. In order to illustrate the effect of the spherical harmonics in this model, the function values of the first spherical harmonics are visualized on the fitted ellipsoid as a spherical reference body in Fig. 3.

### Evaluation of the plagiocephaly-induced shape

The model described in Eq. (1) is used to reconstruct all cranial 3D models with l_max_ values ranging from l_max_ = 2 to l_max_ = 10.

For the detection of DP, it is not required to reconstruct the fine details of the cranial shape. Instead, the head shape abnormality caused by plagiocephaly should be reflected in the first spherical harmonics, as it is commonly defined by an asymmetric flattening of the back of the head. The spherical harmonic of degree l = 2 and order m = − 2 displayed in Fig. 3f is the most promising one to reflect information on plagiocephaly.

This spherical harmonic model divides the head 3D model into four quadrants, where two quadrants approximately form the back and the other two the front of the head. Every two quadrants have inverse function values of the spherical harmonic ${Y}_{2}^{-2}$. In consequence, this spherical harmonic will result in higher coefficients when one of the quadrant head has positive differences and the opposite one has negative differences.

## Results

With the presented approach given in Eq. (1), all cranial 3D models could be reconstructed with a small error with only small maximum spherical harmonic degrees. With a l_max_ = 4 the root-mean-square error (RMSE) of the model was below 1 mm for all 18 heads.

**Figure 4:**
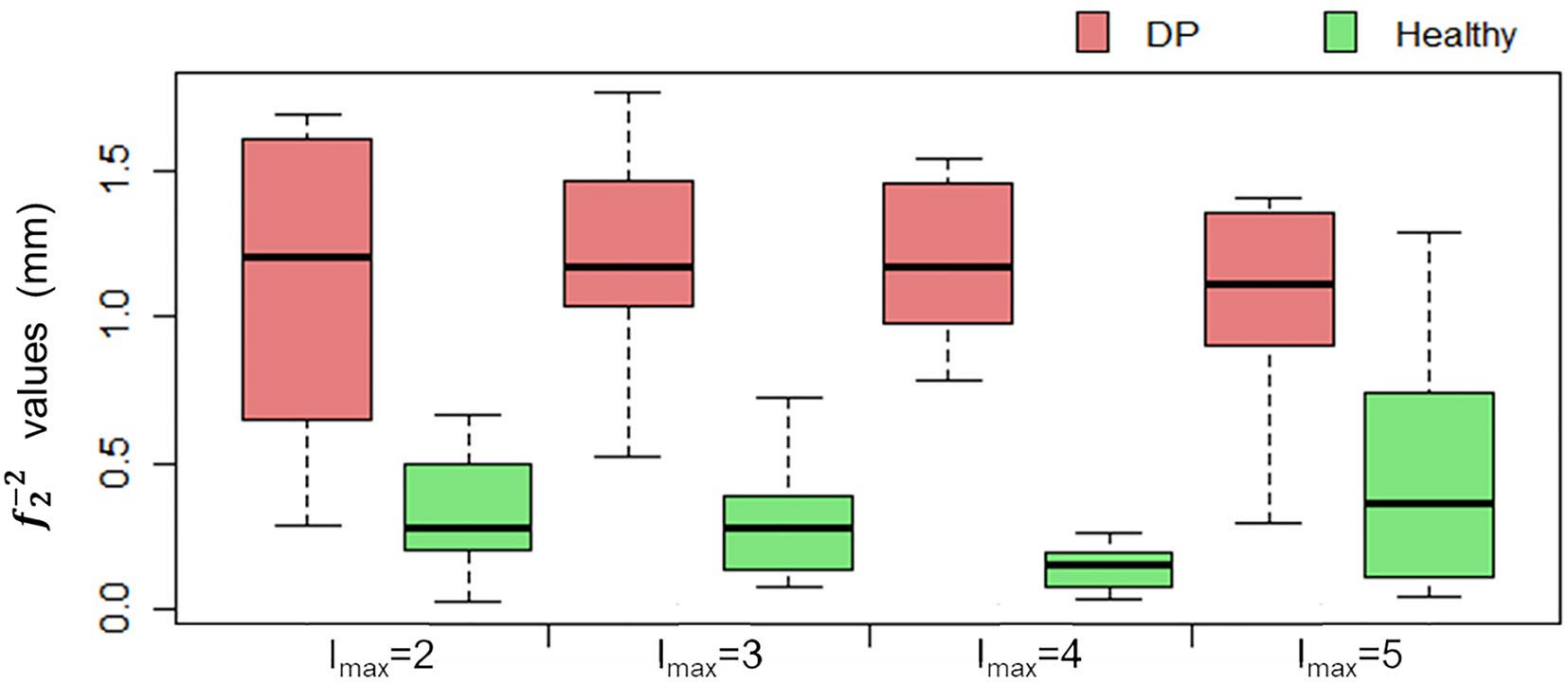
Boxplots of the absolute values of ${f}_{2}^{-2}$ calculated with different lmax values for the two groups of regular heads and heads with DP.

The differences in coefficients for the spherical harmonics of degree l = 2 and order m = − 2 and different values of l_max_ are shown in Fig. 4. The largest difference between healthy patients and DP affected patients is found for l_max_ = 4. For other values of l_max_, the differences are considerably lower.

In order to carry out the statistical analysis, all coefficients and indexes were used in absolute values.

**Figure 5:**
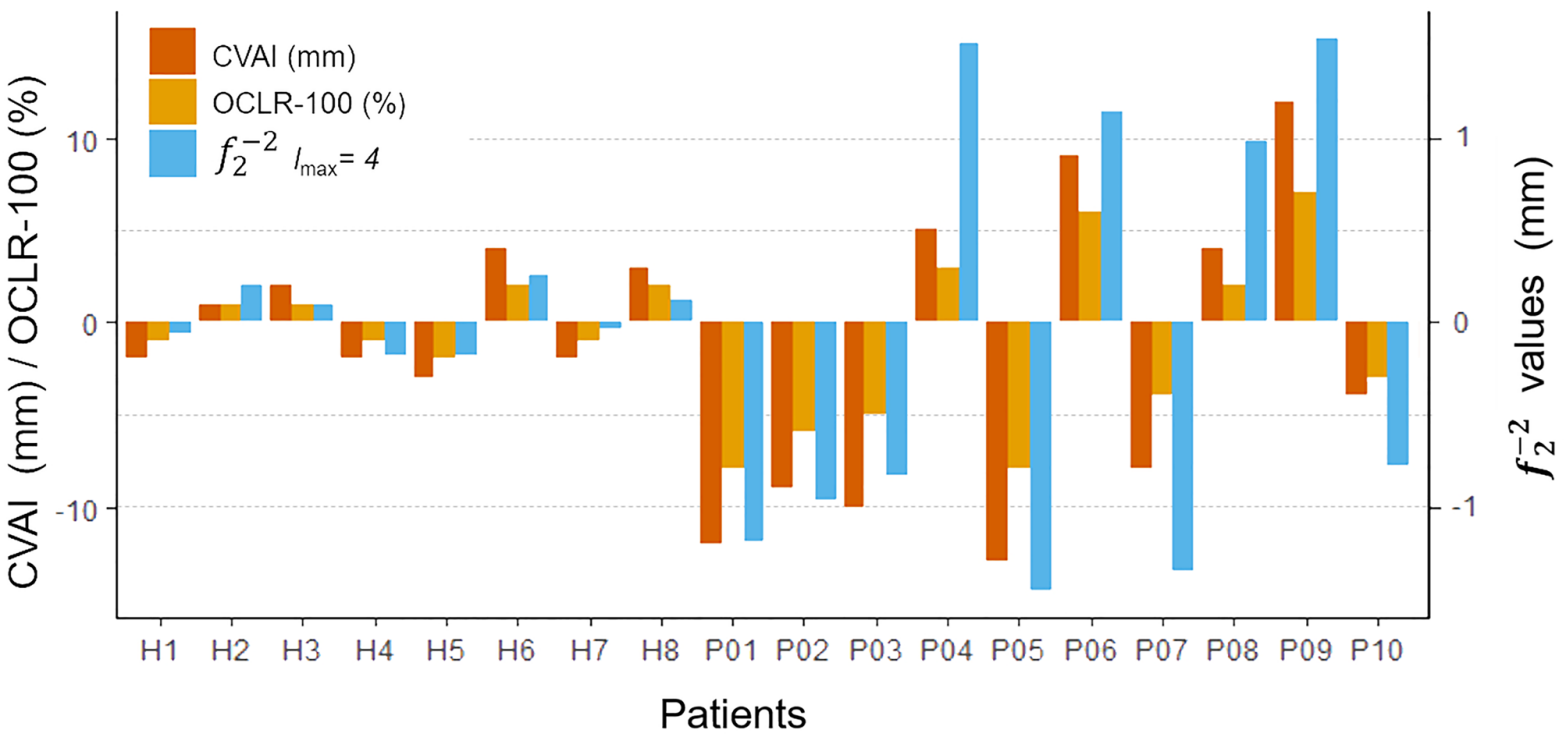
Values of asymmetry index, and normalized OCLR and coefficients of ${f}_{2}^{-2}$ lmax = 4 for the two groups of healthy heads (H) and heads with DP (P). Negative and positive values indicate right and left side plagiocephaly (depressed back side area of the head) respectively.

There exists an important correlation between the values of AI, OCLR, and ${f}_{2}^{-2}$ lmax = 4. In order to be able to compare the different parameters, the spherical harmonic coefficients of each 3D model have been multiplied 10 times (10x) and the OCLR has been normalized so a normal head would have an index of zero, yielding the new index OCLR-100. (Fig. 5).

**Figure 6:**
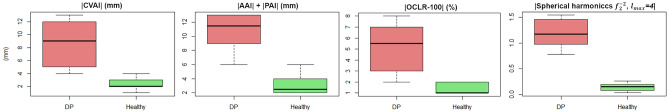
Boxplots of the absolute values of different deformation indexes and coefficient ${f}_{2}^{-2}$ lmax = 4 for the two groups of regular heads and heads with DP.

The objective of this study was to evaluate the capacity of spherical harmonics to detect and quantify DP in relation to conventional and extended anthropomorphic indexes. The distribution of the values of different indexes and coefficients for both groups (regular and with DP) is shown in Fig. 6.

In order to determine whether spherical harmonics coefficients perform better than classical indexes for the discrimination of DP, a statistical Student t-test was performed. The normality and homocedasticity of the full sample were checked beforehand. For every parameter, the p-value was below 0.05. This means that, for every parameter, there is a significant difference in means between regular heads and heads affected with DP. However, the spherical harmonics coefficients reported the lowest p-value and can therefore be considered the best parameter to distinguish between categories. The second best parameter is the sum of the absolute values for anterior and posterior asymmetry indexes. The most commonly used indexes (Asymmetry Index and OCLR) performed considerably worse (Table 2).

**Table 2:** p-values for the different coefficients and indexes computed ordered from highest to lowest confidence.

Coefficient	p-value
|$${f}_{2}^{-2}$$ l_max_ = 4|	3.27E−07
|AAI| +|PAI|	8.67E−07
|$${f}_{2}^{-2}$$ l_max_ = 3|	1.26E−05
|AI|	1.72E−04
|OCLR-100|	2.61E−04
|$${f}_{2}^{-2}$$ l_max_ = 2|	1.14E−03
|$${f}_{2}^{-2}$$ l_max_ = 5|	6.46E−03

**Figure 7:**
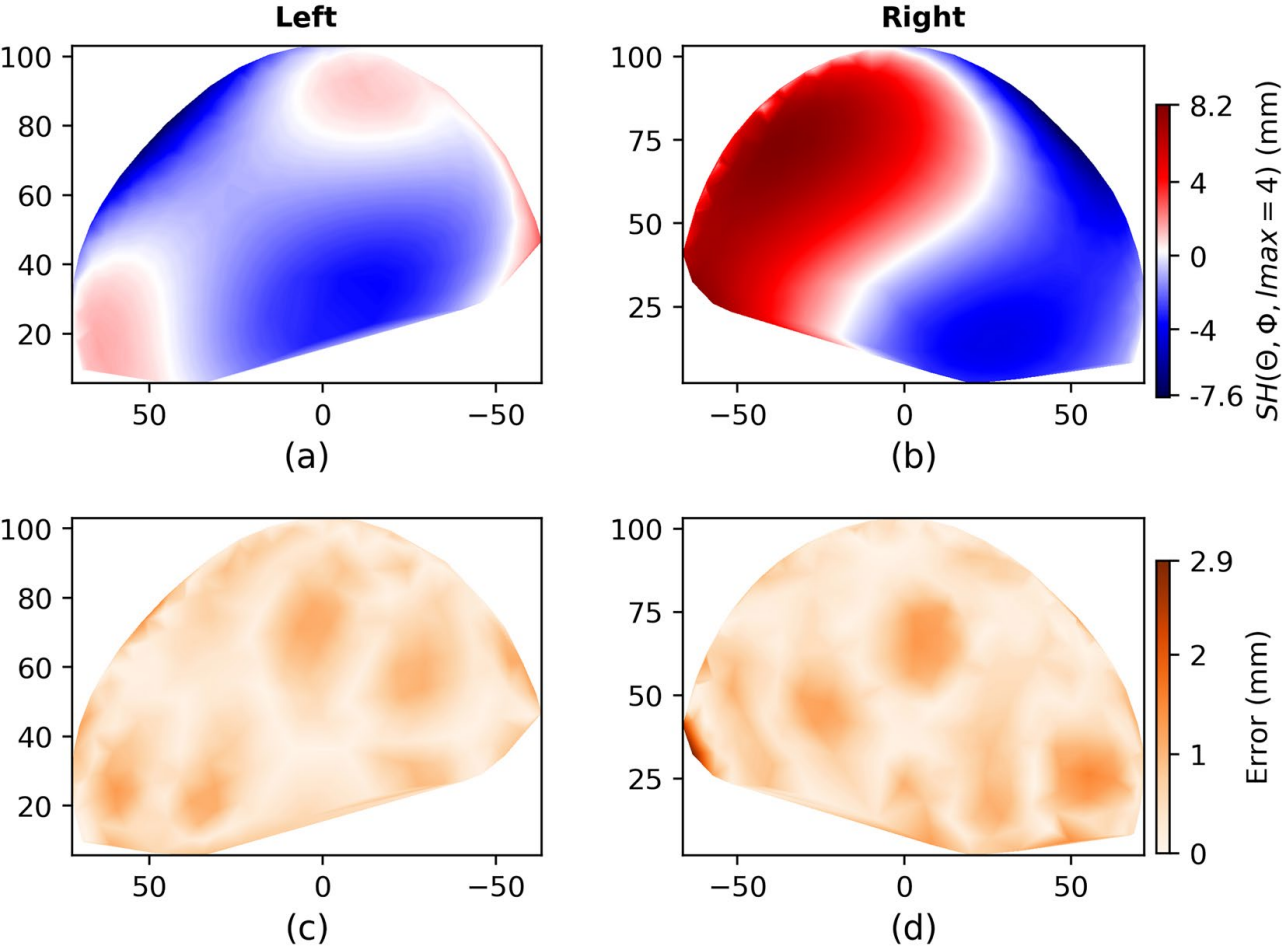
Patient P01: (**a**,**b**) are estimated differences to the fitted ellipsoid with spherical harmonics; (**c**,**d**) difference errors between the original photogrammetric 3D model and the spherical harmonics-based reconstruction. The RMSE for this patient was 0.62 mm.

To check the potential of using spherical harmonics to fit any irregular surface, i.e. heads with DP, Fig. 7 displays the result of lmax = 4 for a the patients P01.

## Discussion

3D models are becoming an important tool to detect and quantify DP and other types of cranial deformations. The models provide a much higher quantity of information in comparison with traditional measurements. Nevertheless, the 3D models should not only be used to compute traditionally simple indexes, because the richness of information is paramount. It is necessary to develop better indexes and coefficients that use all the data contained in the 3D model to allow paediatricians and neurosurgeons assessing the right diagnostics^15,42^.

The spherical harmonic coefficient ${f}_{2}^{-2}$ lmax = 4 is presented as the best option to detect and quantify DP. Even ${f}_{2}^{-2}$ lmax = 3 is better than the |AI|, while the latter outperforms ${f}_{2}^{-2}$ lmax = 2. The assigned weight of the coefficient ${f}_{2}^{-2}$ lmax = 4 has been found to allow better discrimination between DP and regular heads in comparison with traditional measurements.

The threshold for the coefficient ${f}_{2}^{-2}$ lmax = 4 to discriminate regular and DP affected heads is set to 0.42. However, an improvement of this threshold could be carried out by using a larger set of data.

The second-best parameter was the sum of anterior and posterior asymmetry indexes (|AAI| +|PAI|). These parameters have to be extracted from the 3D model as they cannot be computed from traditional analogue measurements taken with a calliper. Without any doubt, the traditional measurements provide considerably worse results to assess the cranial deformation in patients.

Similarly as was demonstrated previously by other authors, increasing lmax up to 8 and upwards, the RMSE converges towards zero, starting from a RMSE value close to 2 mm for lmax = 1. However, as stated above, the maximum differentiation in absolute values of *f*_2_^–2^ is maximum for lmax = 4. Minimizing the RMSE with higher lmax degrees, confirms why other researchers opt for using maximum l-values of 100^43^, 1024^29^ or even an infinite set of harmonic functions^26,27^. Our understanding for reaching a global complexity value, instead of regional or local complexities^29^, confirms the expections of differentiating between regular heads and heads with DP using a relatively low lmax value of 4.

This research opens the door to the future application of spherical harmonics to identify different cranial deformation diseases using different degrees and orders. For instance, other types of deformation in infants such as brachycephaly, scaphocephaly or other types of craniosynostosis will be investigated in the future to confirm the successful implementation of spherical harmonics to help to improve infant’s diagnostics.

Although photogrammetric 3D models of the head has been used for this study, the methodology can also be applied to 3D models obtained by advanced radiological devices such as CT and MRI or high-end photogrammetric/laser scanning solutions. In spite of the fact that CT and MRI are considered the gold standard for diagnostics and surgery intervention, derived measurements are usually obtained manually by radiologists. Because of this, it is required to improve and develop new, mathematically validated anthropometric indexes, that provide useful and accurate information to specialised doctors^14,42^.

## Conclusions

Spherical harmonics are presented as a valuable tool for the detection and measurement of DP, which is a common infant’s disease that requires the right diagnostics in the early life stages. In the present study, spherical harmonics have been first used to assess DP in infants in a simple way. In particular, the spherical harmonics are used to model the distances from the real head to an ideally fitted ellipsoid. The spherical harmonic coefficient of degree 2, order—2 with l_max_ = 4 has been found to provide the best results to discriminate between regular and DP affected heads. This spherical harmonic coefficient has been found to provide better results than the commonly used anthropometric indexes such as AI and OCLR, to discriminate between regular and DP affected heads.

A threshold for ${f}_{2}^{-2}$ lmax = 4 of 0.42 is proposed in order to discriminate between regular and DP affected heads.

Moreover, it has been found that the simple sum of the absolute values for anterior and posterior asymmetry indexes (|AAI| +|PAI|) performs better than AI and OCLR, independently of whether these two latter indexes are extracted from the 3D model, the 2D images from CT and MRI. However, AAI and PAI cannot be determined by using a calliper/cephalometer, despite its easy computation.

In the near future, further research will be devoted to assessing the performance of spherical harmonics to assess additional cranial deformation problems such as brachycephaly, scaphocephaly, hydrocephaly and craniosynostosis. Last but not least, the presented threshold will be refined with a larger dataset.
